# (2-Amino­cyclo­hexanol-κ^2^*N*,*O*)chlorido(η^6^-*p*-cy­mene)ru­thenium(II) tetra­fluoro­borate (2-amino­cyclo­hexanolato-κ^2^*N*,*O*)chlorido(η^6^-*p*-cymene)ru­thenium(II)

**DOI:** 10.1107/S2414314626006103

**Published:** 2026-06-16

**Authors:** Michael Oerder, Glenn E.M. Maguire, Hendirk G. Kruger, Sizwe J. Zamisa

**Affiliations:** a Catalysis and Peptide Research Unit, University of KwaZulu-Natal, Private Bag X54001, Durban 4000, South Africa; b School of Agriculture and Science, Discipline of Chemistry, University of KwaZulu-Natal, Private Bag X54001, Durban 4000, South Africa; Howard University, USA

**Keywords:** crystal structure, Ru^II^ complex, BF_4_^−^, 2-amino-cyclo­hexa­nol

## Abstract

The asymmetric unit of the title compound, [RuCl(C_6_H_13_NO)(C_10_H_14_)]BF_4_·[Ru Cl(C_10_H_14_)(C_6_H_12_NO], crystallizes with one neutral Ru^II^ species, one cationic Ru^II^ species and a BF_4_^−^ counter-ion. Both Ru^II^ centres adopt the expected piano-stool geometry, with the η^6^-*p*-cymene ligand forming the seat and the chlorido and bidentate amino alcohol-derived ligands occupying the remaining coordination sites. The steric influence of the cyclo­hexyl-based ligand framework is reflected in a slightly longer O⋯O contact [2.448 (4) Å] than that observed in the closely related 2-amino­ethano­late analogue [2.397 (7) Å; Tse *et al.* (2011 ▸). *Chem. Eur, J.***17**, 13918–13925.]. In the crystal, alternating N—H⋯F and O—H⋯O hydrogen-bonding inter­actions link the components into a supra­molecular chain extending along the *c-*axis direction.

## Abstract 2

The title compound crystallizes with one neutral Ru^II^ species, one cationic Ru^II^ species and a BF_4_^−^ counter-ion in the asymmetric unit. Both Ru^II^ centres adopt the expected piano-stool geometry, with the η^6^-*p*-cymene ligand forming the seat and the chlorido and bidentate amino-alcohol-derived ligands occupying the remaining coordination sites.

## Graphical abstract

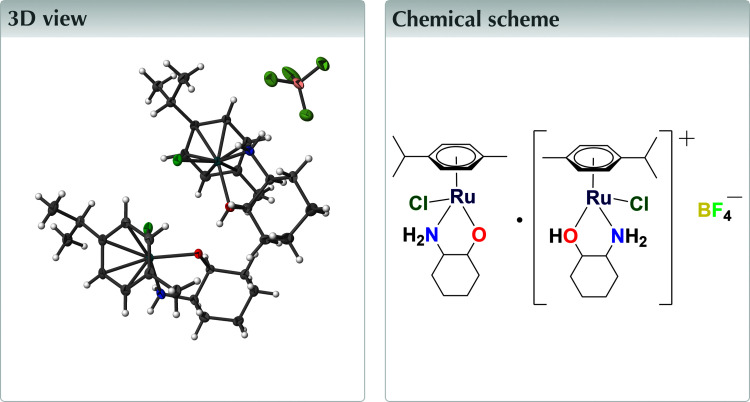

## Structure description

Piano-stool Ru^II^ complexes are of continuing inter­est because of their catalytic and medicinal relevance (Sojka & Gamez, 2025 ▸; Kumah & Ojwach, 2023 ▸). Most reported examples contain *N*,*N*- or *N*,*O*-bidentate ligands, whereas structurally characterized amino alcohol/chlorido analogues remain relatively uncommon (Kumar *et al.*, 2014 ▸). The closest related structure is [μ_2_-hydrogenbis(2-amino­ethano­late)]di­chloro­bis­(η^6^-*p*-cymene)diruthenium chloride aceto­nitrile solvate (CSD refcode: RAKNUO; Tse *et al.*, 2011 ▸), in which a short O⋯O contact of 2.397 (7) Å was attributed to strong hydrogen bonding between neighbouring 2-amino­ethano­late ligands. In this work, replacement of the 2-amino­ethanol ligand by the more sterically demanding 2-amino­cyclo­hexa­nol is studied.

**Figure 1:**
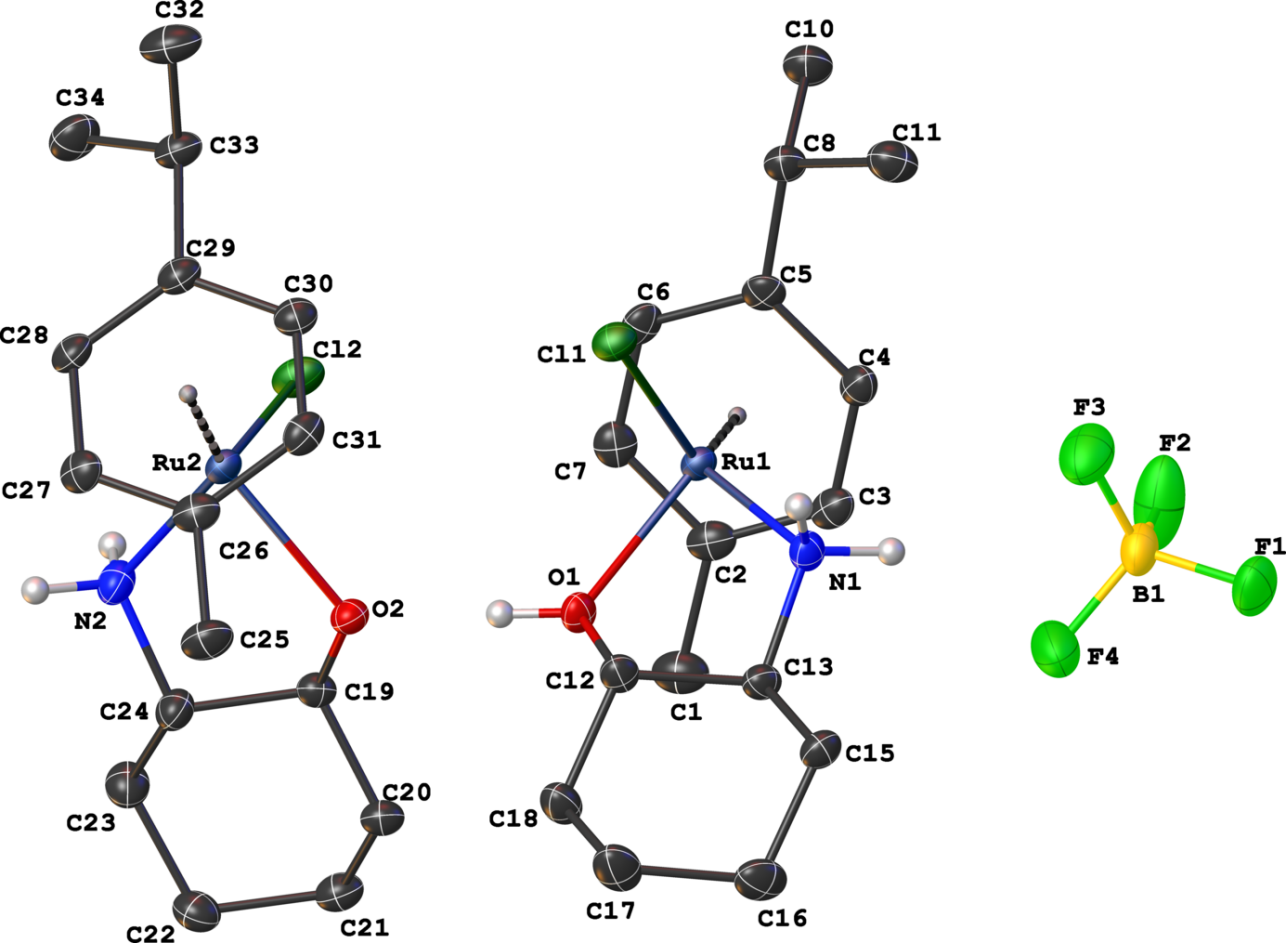
Mol­ecular structure of the title compound showing the atom-numbering scheme and displacement ellipsoids drawn at the 50% probability level. Only the polar hydrogen atoms are shown for clarity.

**Figure 2:**
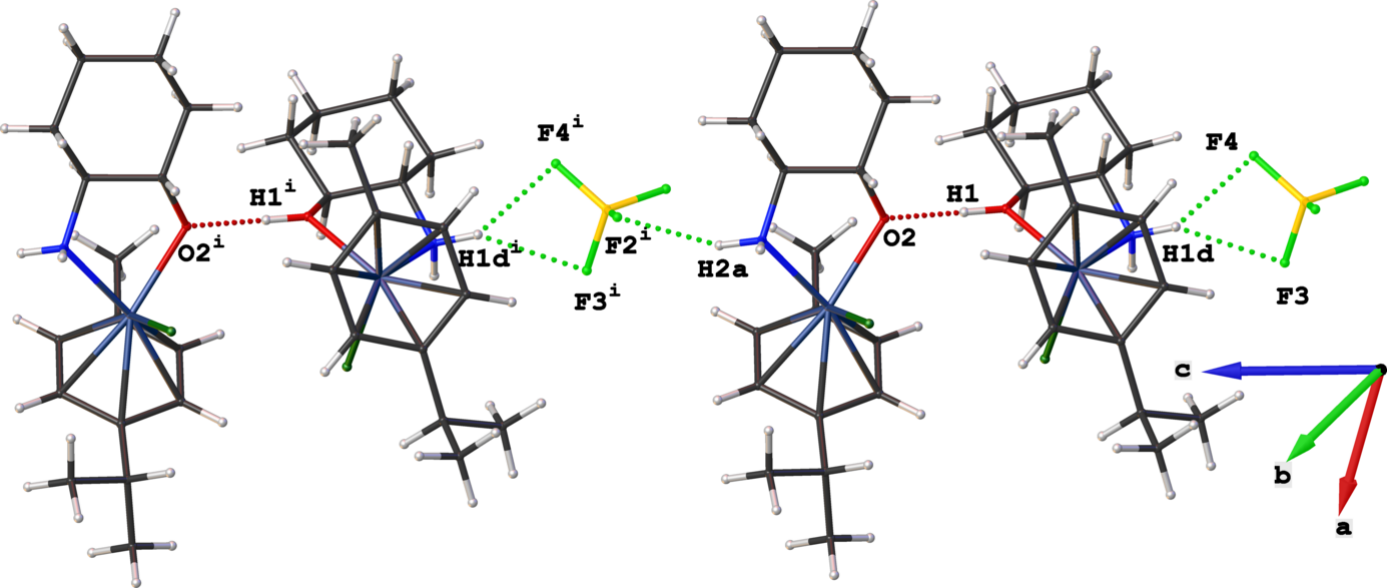
Representation of the inter­molecular O—H⋯O and N—H⋯F hydrogen bonds in the crystal structure of the title compound.

The asymmetric unit of the title compound contains one neutral Ru^II^ complex and one cationic Ru^II^ complex with a BF_4_^−^ counter-ion (Fig. 1 ▸). Both Ru^II^ centres adopt the expected piano-stool geometry, with the η^6^-*p*-cymene ligand forming the seat and the chlorido and bidentate amino-alcohol-derived ligands forming the legs. The neutral complex contains a 2-amino­cyclo­hexa­nolate ligand, whereas the cationic complex contains a 2-amino­cyclo­hexa­nol ligand. Bond lengths and angles are comparable with those observed in RAKNUO, although the O⋯O separation is slightly longer at 2.448 (4) Å, consistent with the greater steric demand of the cyclo­hexyl group relative to the ethyl­ene fragment in the related structure. In the crystal packing of the title compound, alternating inter­molecular N—H⋯F and O—H⋯O hydrogen-bonding patterns (Table 1 ▸) form a supra­molecular chain that extends along the crystallographic *c-*axis direction (Fig. 2 ▸).

## Synthesis and crystallization

Di­ethyl­amino­methyl­polystyrene (0.100 g, 0.327 mmol,) was added to a round-bottom flask containing 15 ml of dry di­chloro­methane. The amino alcohol (1 mol eq.) was added and allowed to stir at room temperature for 15 minutes. Ruthenium dimer [(η^6^-ρ-cymene)RuCl_2_]_2_ (0.5 mol eq, 0.100 g, 0.163 mmol) was added to the reaction and the mixture was allowed to stir for 24 h at room temperature. The complex was isolated by evaporating under reduced pressure using a rotary evaporator. The purification of the ruthenium complex was performed by allowing it to stir in diethyl ether for 24 h. The solid product was filtered off under vacuum. 100 mg of the complex were dissolved in methanol (10 mL) and one molar equivalent of sodium tetra­fluoro­borate added at room temperature. The mixture was allowed to stir for 1 h, after which excess salt was vacuum filtered. After two days a mixture of crystalline salt and complex crystals developed from solution. The complex was soluble in THF while the salt crystals were not, so THF was added dropwise until the complex was all dissolved. This was filtered and the filtrate was allowed to evaporate. The resulting solid was dissolved in acetone (12 mL). An open vial was sealed in a larger vial containing diethyl ether, allowing for vapour diffusion to promote crystal growth.

## Refinement

Crystal data, data collection, and structure refinement details are summarized in Table 2 ▸.

## Supplementary Material

Crystal structure: contains datablock(s) I. DOI: 10.1107/S2414314626006103/bv4060sup1.cif

Structure factors: contains datablock(s) I. DOI: 10.1107/S2414314626006103/bv4060Isup2.hkl

CCDC reference: 2561435

Additional supporting information:  crystallographic information; 3D view; checkCIF report

## Figures and Tables

**Table 1:** Hydrogen-bond geometry (Å, °)

*D*—H⋯*A*	*D*—H	H⋯*A*	*D*⋯*A*	*D*—H⋯*A*
N1—H1*D*⋯F3	0.91	2.33	3.150 (3)	150
N1—H1*D*⋯F4	0.91	2.21	3.032 (3)	150
N2—H2*A*⋯F2^i^	0.91	2.15	2.996 (3)	154
O1—H1⋯O2	0.84 (1)	1.61 (1)	2.449 (2)	176 (4)

Symmetry code: (i) 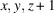.

**Table 2:** Experimental details

Crystal data	
Chemical formula	[RuCl(C_6_H_13_NO)(C_10_H_14_)]BF_4_·[Ru Cl(C_10_H_14_)(C_6_H_12_NO]
*M*_r_	857.61
Crystal system, space group	Triclinic, *P* 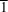
Temperature (K)	100
*a*, *b*, *c* (Å)	12.2424 (11), 13.8251 (13), 13.9939 (13)
α, β, γ (°)	60.974 (2), 72.960 (3), 67.976 (2)
*V* (Å^3^)	1901.8 (3)
*Z*	2
Radiation type	Mo *K*α
μ (mm^−1^)	0.98
Crystal size (mm)	0.26 × 0.14 × 0.09

Data collection	
Diffractometer	Bruker SMART APEXII area detector
Absorption correction	Multi-scan (*SADABS*; Krause *et al.*, 2015 ▸)
*T*_min_, *T*_max_	0.775, 0.926
No. of measured, independent and observed [*I* > 2σ(*I*)] reflections	53489, 8829, 7079
*R*_int_	0.047
(sin θ/λ)_max_ (Å^−1^)	0.655

Refinement	
*R*[*F*^2^ > 2σ(*F*^2^)], *wR*(*F*^2^), *S*	0.034, 0.091, 1.06
No. of reflections	8829
No. of parameters	411
No. of restraints	1
H-atom treatment	H atoms treated by a mixture of independent and constrained refinement
Δρ_max_, Δρ_min_ (e Å^−3^)	1.11, −1.04

Computer programs: *APEX2*, *COSMO* and *SAINT* (Bruker, 2009 ▸), *SHELXS2013* (Sheldrick, 2008 ▸), *SHELXL2018/3* (Sheldrick, 2015 ▸) and *OLEX2* (Dolomanov *et al.*, 2009 ▸).

## full crystallographic data

### (2-Aminocyclohexanol-κ2N,O)chlorido(η6-p-cymene)ruthenium(II) tetrafluoroborate (2-aminocyclohexanolato-κ2N,O)chlorido(η6-p-cymene)ruthenium(II) Crystal data

**Table:**

[RuCl(C_6_H_13_NO)(C_10_H_14_)]BF_4_·[RuCl(C_10_H_14_)(C_6_H_12_NO)]	*Z* = 2
*M**_r_* = 857.61	*F*(000) = 876
Triclinic, *P*1	*D*_x_ = 1.498 Mg m^−^^3^
*a* = 12.2424 (11) Å	Mo *K*α radiation, λ = 0.71073 Å
*b* = 13.8251 (13) Å	Cell parameters from 9804 reflections
*c* = 13.9939 (13) Å	θ = 2.3–27.6°
α = 60.974 (2)°	µ = 0.98 mm^−^^1^
β = 72.960 (3)°	*T* = 100 K
γ = 67.976 (2)°	Block, orange
*V* = 1901.8 (3) Å^3^	0.26 × 0.14 × 0.09 mm

### (2-Aminocyclohexanol-κ2N,O)chlorido(η6-p-cymene)ruthenium(II) tetrafluoroborate (2-aminocyclohexanolato-κ2N,O)chlorido(η6-p-cymene)ruthenium(II) Data collection

**Table:**

Bruker SMART APEXII area detector diffractometer	8829 independent reflections
Radiation source: microfocus sealed X-ray tube, Incoatec Iµs	7079 reflections with *I* > 2σ(*I*)
Graphite monochromator	*R*_int_ = 0.047
Detector resolution: 7.9 pixels mm^-1^	θ_max_ = 27.8°, θ_min_ = 1.7°
ω and φ scans	*h* = −15→15
Absorption correction: multi-scan (SADABS; Krause *et al.*, 2015)	*k* = −18→17
*T*_min_ = 0.775, *T*_max_ = 0.926	*l* = −18→18
53489 measured reflections	

### (2-Aminocyclohexanol-κ2N,O)chlorido(η6-p-cymene)ruthenium(II) tetrafluoroborate (2-aminocyclohexanolato-κ2N,O)chlorido(η6-p-cymene)ruthenium(II) Refinement

**Table:**

Refinement on *F*^2^	1 restraint
Least-squares matrix: full	Hydrogen site location: mixed
*R*[*F*^2^ > 2σ(*F*^2^)] = 0.034	H atoms treated by a mixture of independent and constrained refinement
*wR*(*F*^2^) = 0.091	*w* = 1/[σ^2^(*F*_o_^2^) + (0.0513*P*)^2^ + 0.5637*P*] where *P* = (*F*_o_^2^ + 2*F*_c_^2^)/3
*S* = 1.06	(Δ/σ)_max_ = 0.003
8829 reflections	Δρ_max_ = 1.11 e Å^−^^3^
411 parameters	Δρ_min_ = −1.04 e Å^−^^3^

### (2-Aminocyclohexanol-κ2N,O)chlorido(η6-p-cymene)ruthenium(II) tetrafluoroborate (2-aminocyclohexanolato-κ2N,O)chlorido(η6-p-cymene)ruthenium(II) Special details

**Table:**

Geometry. All esds (except the esd in the dihedral angle between two l.s. planes) are estimated using the full covariance matrix. The cell esds are taken into account individually in the estimation of esds in distances, angles and torsion angles; correlations between esds in cell parameters are only used when they are defined by crystal symmetry. An approximate (isotropic) treatment of cell esds is used for estimating esds involving l.s. planes.
Refinement. All aromatic, methyl, methylene and methine hydrogen atoms were placed in idealized positions and refined in riding models with their respective parent atoms. The C—H distances were constrained to 0.95 Å for all the aromatic H atoms, 0.98 Å for methyl hydrogens, 0.99 Å for methylene hydrogens, 1.00 Å and 0.91 Å for amine hydrogens. The hydroxyl hydrogen atom was located and positioned geometrically with O—H distance constrained to 0.84 Å.

### (2-Aminocyclohexanol-κ2N,O)chlorido(η6-p-cymene)ruthenium(II) tetrafluoroborate (2-aminocyclohexanolato-κ2N,O)chlorido(η6-p-cymene)ruthenium(II) Fractional atomic coordinates and isotropic or equivalent isotropic displacement parameters (Å^2^)

**Table:**

	*x*	*y*	*z*	*U*_iso_*/*U*_eq_	
C1	−0.0538 (2)	0.8003 (2)	0.4942 (2)	0.0271 (6)	
H1A	−0.070345	0.826795	0.551811	0.041*	
H1B	−0.121899	0.837814	0.453178	0.041*	
H1C	−0.040104	0.716652	0.528112	0.041*	
C2	0.0546 (2)	0.8306 (2)	0.4168 (2)	0.0213 (6)	
C3	0.0988 (2)	0.8027 (2)	0.3261 (2)	0.0212 (6)	
H3	0.055523	0.769319	0.309790	0.025*	
C4	0.2070 (2)	0.8235 (2)	0.2579 (2)	0.0199 (5)	
H4	0.234660	0.803796	0.196985	0.024*	
C5	0.2737 (2)	0.8728 (2)	0.2794 (2)	0.0186 (5)	
C6	0.2278 (2)	0.9039 (2)	0.3700 (2)	0.0195 (5)	
H6	0.270188	0.939187	0.384857	0.023*	
C7	0.1217 (2)	0.8836 (2)	0.4372 (2)	0.0212 (6)	
H7	0.093353	0.904890	0.497051	0.025*	
C8	0.3940 (2)	0.8886 (2)	0.2139 (2)	0.0202 (5)	
H8	0.445442	0.873067	0.266453	0.024*	
C10	0.3775 (3)	1.0152 (2)	0.1286 (2)	0.0270 (6)	
H10A	0.454678	1.026142	0.085998	0.040*	
H10B	0.323239	1.034425	0.078600	0.040*	
H10C	0.344017	1.065618	0.166898	0.040*	
C11	0.4581 (3)	0.8065 (2)	0.1584 (2)	0.0273 (6)	
H11A	0.534651	0.821227	0.117805	0.041*	
H11B	0.471717	0.726796	0.214717	0.041*	
H11C	0.409104	0.818977	0.107102	0.041*	
C12	0.2361 (2)	0.4978 (2)	0.6201 (2)	0.0185 (5)	
H12	0.323241	0.470028	0.625086	0.022*	
C13	0.2174 (2)	0.4872 (2)	0.5239 (2)	0.0183 (5)	
H13	0.130734	0.520834	0.516491	0.022*	
C15	0.2527 (2)	0.3634 (2)	0.5390 (2)	0.0199 (5)	
H15A	0.339852	0.330388	0.538752	0.024*	
H15B	0.231669	0.361428	0.476965	0.024*	
C16	0.1887 (3)	0.2915 (2)	0.6480 (2)	0.0280 (6)	
H16A	0.102071	0.319372	0.644978	0.034*	
H16B	0.216487	0.210027	0.659549	0.034*	
C17	0.2131 (3)	0.3002 (2)	0.7444 (2)	0.0304 (7)	
H17A	0.299291	0.268292	0.750035	0.036*	
H17B	0.170242	0.254172	0.814107	0.036*	
C18	0.1724 (3)	0.4259 (2)	0.7276 (2)	0.0239 (6)	
H18A	0.085435	0.456613	0.726015	0.029*	
H18B	0.189856	0.430383	0.789846	0.029*	
C19	0.0631 (2)	0.7089 (2)	0.7866 (2)	0.0172 (5)	
H19	0.035778	0.790183	0.731238	0.021*	
C20	−0.0220 (2)	0.6435 (2)	0.8026 (2)	0.0193 (5)	
H20A	0.004866	0.562445	0.856272	0.023*	
H20B	−0.021918	0.644027	0.731626	0.023*	
C21	−0.1476 (2)	0.6994 (2)	0.8448 (2)	0.0230 (6)	
H21A	−0.201913	0.655878	0.855619	0.028*	
H21B	−0.175851	0.779155	0.789084	0.028*	
C22	−0.1504 (2)	0.7015 (2)	0.9536 (2)	0.0243 (6)	
H22A	−0.130351	0.621639	1.011292	0.029*	
H22B	−0.231669	0.742131	0.976951	0.029*	
C23	−0.0620 (2)	0.7623 (3)	0.9416 (2)	0.0249 (6)	
H23A	−0.088756	0.845108	0.892105	0.030*	
H23B	−0.059485	0.755706	1.014661	0.030*	
C24	0.0609 (2)	0.7096 (2)	0.8951 (2)	0.0210 (6)	
H24	0.088399	0.627855	0.949537	0.025*	
C25	0.3847 (3)	0.4385 (2)	0.9203 (2)	0.0258 (6)	
H25A	0.333675	0.433243	0.881530	0.039*	
H25B	0.341586	0.436548	0.992004	0.039*	
H25C	0.456670	0.373395	0.931450	0.039*	
C26	0.4187 (2)	0.5495 (2)	0.8525 (2)	0.0194 (5)	
C27	0.4123 (2)	0.6204 (2)	0.9016 (2)	0.0208 (6)	
H27	0.389597	0.595815	0.978968	0.025*	
C28	0.4393 (2)	0.7285 (2)	0.8368 (2)	0.0196 (5)	
H28	0.436050	0.774063	0.871558	0.024*	
C29	0.4709 (2)	0.7678 (2)	0.7215 (2)	0.0197 (5)	
C30	0.4774 (2)	0.6963 (2)	0.6720 (2)	0.0198 (5)	
H30	0.500300	0.720584	0.594686	0.024*	
C31	0.4503 (2)	0.5905 (2)	0.7363 (2)	0.0194 (5)	
H31	0.453233	0.545222	0.701336	0.023*	
C32	0.6339 (3)	0.8599 (3)	0.6202 (3)	0.0314 (7)	
H32A	0.653570	0.932951	0.571785	0.047*	
H32B	0.667979	0.823972	0.688902	0.047*	
H32C	0.666903	0.808259	0.583193	0.047*	
C33	0.4984 (2)	0.8823 (2)	0.6465 (2)	0.0216 (6)	
H33	0.467164	0.913284	0.575662	0.026*	
C34	0.4411 (3)	0.9734 (3)	0.6921 (3)	0.0302 (7)	
H34A	0.462885	1.044465	0.639254	0.045*	
H34B	0.354364	0.988879	0.703988	0.045*	
H34C	0.469133	0.945082	0.762299	0.045*	
N1	0.2826 (2)	0.56080 (18)	0.42479 (17)	0.0181 (5)	
H1D	0.261916	0.570065	0.362848	0.022*	
H1E	0.362292	0.527008	0.424266	0.022*	
N2	0.14981 (19)	0.7688 (2)	0.87582 (19)	0.0216 (5)	
H2A	0.171049	0.749077	0.941404	0.026*	
H2B	0.117036	0.846417	0.844630	0.026*	
O1	0.19493 (16)	0.61792 (15)	0.59536 (15)	0.0177 (4)	
O2	0.18286 (15)	0.66000 (15)	0.74975 (14)	0.0172 (4)	
B1	0.1951 (3)	0.6071 (3)	0.1731 (3)	0.0277 (7)	
F1	0.20077 (19)	0.54198 (17)	0.12112 (15)	0.0415 (5)	
F2	0.1359 (2)	0.72104 (18)	0.11141 (18)	0.0593 (7)	
F3	0.30738 (17)	0.60379 (19)	0.17774 (17)	0.0449 (5)	
F4	0.13506 (19)	0.5695 (2)	0.27905 (16)	0.0506 (6)	
Cl1	0.44144 (6)	0.65903 (6)	0.46189 (5)	0.02135 (14)	
Cl2	0.21343 (6)	0.90927 (6)	0.63402 (6)	0.02463 (15)	
Ru1	0.23903 (2)	0.72358 (2)	0.42743 (2)	0.01525 (7)	
Ru2	0.30306 (2)	0.72205 (2)	0.77018 (2)	0.01533 (7)	
H1	0.189 (4)	0.630 (3)	0.6502 (16)	0.079 (15)*	

### (2-Aminocyclohexanol-κ2N,O)chlorido(η6-p-cymene)ruthenium(II) tetrafluoroborate (2-aminocyclohexanolato-κ2N,O)chlorido(η6-p-cymene)ruthenium(II) Atomic displacement parameters (Å^2^)

**Table:**

	*U*^11^	*U*^22^	*U*^33^	*U*^12^	*U*^13^	*U*^23^
C1	0.0207 (14)	0.0216 (15)	0.0295 (15)	−0.0047 (11)	−0.0059 (12)	−0.0033 (12)
C2	0.0176 (13)	0.0153 (13)	0.0250 (14)	−0.0025 (10)	−0.0076 (11)	−0.0029 (11)
C3	0.0222 (13)	0.0179 (14)	0.0218 (13)	−0.0060 (11)	−0.0111 (11)	−0.0026 (11)
C4	0.0247 (14)	0.0187 (13)	0.0165 (12)	−0.0078 (11)	−0.0072 (11)	−0.0039 (11)
C5	0.0240 (14)	0.0094 (12)	0.0174 (12)	−0.0059 (10)	−0.0053 (11)	0.0004 (10)
C6	0.0246 (14)	0.0120 (12)	0.0237 (13)	−0.0047 (10)	−0.0068 (11)	−0.0074 (11)
C7	0.0234 (14)	0.0138 (13)	0.0226 (13)	−0.0004 (10)	−0.0064 (11)	−0.0065 (11)
C8	0.0224 (13)	0.0183 (13)	0.0200 (13)	−0.0089 (11)	−0.0013 (11)	−0.0067 (11)
C10	0.0308 (16)	0.0238 (15)	0.0254 (14)	−0.0126 (12)	−0.0015 (12)	−0.0073 (12)
C11	0.0291 (15)	0.0243 (15)	0.0258 (15)	−0.0112 (12)	0.0014 (12)	−0.0087 (12)
C12	0.0229 (13)	0.0140 (13)	0.0185 (12)	−0.0058 (10)	−0.0027 (11)	−0.0065 (11)
C13	0.0190 (13)	0.0173 (13)	0.0196 (13)	−0.0070 (10)	−0.0003 (10)	−0.0085 (11)
C15	0.0206 (13)	0.0183 (13)	0.0240 (13)	−0.0073 (11)	−0.0033 (11)	−0.0099 (11)
C16	0.0343 (16)	0.0185 (14)	0.0314 (15)	−0.0115 (12)	0.0018 (13)	−0.0111 (12)
C17	0.0464 (19)	0.0210 (15)	0.0226 (14)	−0.0149 (13)	0.0001 (13)	−0.0071 (12)
C18	0.0319 (15)	0.0215 (14)	0.0172 (13)	−0.0109 (12)	−0.0005 (11)	−0.0065 (11)
C19	0.0170 (12)	0.0165 (13)	0.0175 (12)	−0.0054 (10)	−0.0011 (10)	−0.0068 (11)
C20	0.0179 (13)	0.0172 (13)	0.0214 (13)	−0.0064 (10)	−0.0021 (11)	−0.0062 (11)
C21	0.0198 (13)	0.0214 (14)	0.0274 (14)	−0.0075 (11)	−0.0047 (11)	−0.0079 (12)
C22	0.0198 (13)	0.0272 (15)	0.0222 (14)	−0.0074 (12)	0.0000 (11)	−0.0086 (12)
C23	0.0188 (13)	0.0307 (16)	0.0261 (14)	−0.0048 (12)	−0.0029 (11)	−0.0145 (13)
C24	0.0196 (13)	0.0251 (15)	0.0233 (13)	−0.0068 (11)	−0.0040 (11)	−0.0130 (12)
C25	0.0260 (15)	0.0195 (14)	0.0308 (15)	−0.0095 (12)	−0.0059 (12)	−0.0064 (12)
C26	0.0134 (12)	0.0164 (13)	0.0252 (14)	−0.0016 (10)	−0.0070 (11)	−0.0057 (11)
C27	0.0196 (13)	0.0203 (14)	0.0210 (13)	−0.0040 (11)	−0.0100 (11)	−0.0048 (11)
C28	0.0176 (13)	0.0204 (14)	0.0249 (14)	−0.0066 (10)	−0.0073 (11)	−0.0091 (11)
C29	0.0164 (12)	0.0195 (13)	0.0248 (14)	−0.0062 (10)	−0.0050 (11)	−0.0086 (11)
C30	0.0148 (12)	0.0218 (14)	0.0231 (13)	−0.0053 (10)	−0.0024 (11)	−0.0096 (11)
C31	0.0159 (12)	0.0175 (13)	0.0276 (14)	−0.0005 (10)	−0.0057 (11)	−0.0131 (11)
C32	0.0233 (15)	0.0267 (16)	0.0397 (17)	−0.0120 (12)	−0.0067 (13)	−0.0058 (14)
C33	0.0201 (13)	0.0190 (14)	0.0254 (14)	−0.0083 (11)	−0.0059 (11)	−0.0056 (11)
C34	0.0376 (17)	0.0224 (15)	0.0352 (16)	−0.0130 (13)	−0.0074 (14)	−0.0108 (13)
N1	0.0224 (11)	0.0172 (11)	0.0170 (11)	−0.0059 (9)	−0.0031 (9)	−0.0085 (9)
N2	0.0189 (11)	0.0262 (13)	0.0252 (12)	−0.0057 (10)	−0.0043 (10)	−0.0149 (10)
O1	0.0221 (9)	0.0149 (9)	0.0179 (9)	−0.0046 (7)	−0.0031 (8)	−0.0085 (8)
O2	0.0158 (9)	0.0184 (9)	0.0194 (9)	−0.0062 (7)	−0.0023 (7)	−0.0085 (8)
B1	0.0303 (18)	0.0345 (19)	0.0268 (17)	−0.0085 (15)	−0.0044 (14)	−0.0196 (15)
F1	0.0640 (13)	0.0386 (11)	0.0371 (10)	−0.0223 (10)	−0.0068 (9)	−0.0216 (9)
F2	0.0883 (18)	0.0372 (12)	0.0583 (14)	0.0110 (11)	−0.0438 (13)	−0.0267 (11)
F3	0.0316 (10)	0.0647 (14)	0.0521 (12)	−0.0144 (10)	−0.0015 (9)	−0.0366 (11)
F4	0.0506 (13)	0.0837 (17)	0.0335 (10)	−0.0369 (12)	0.0059 (9)	−0.0300 (11)
Cl1	0.0181 (3)	0.0208 (3)	0.0251 (3)	−0.0061 (3)	−0.0052 (3)	−0.0079 (3)
Cl2	0.0239 (3)	0.0161 (3)	0.0304 (4)	−0.0066 (3)	−0.0103 (3)	−0.0029 (3)
Ru1	0.01634 (11)	0.01394 (12)	0.01664 (11)	−0.00552 (8)	−0.00319 (8)	−0.00589 (9)
Ru2	0.01577 (11)	0.01381 (11)	0.01782 (11)	−0.00476 (8)	−0.00369 (8)	−0.00653 (9)

### (2-Aminocyclohexanol-κ2N,O)chlorido(η6-p-cymene)ruthenium(II) tetrafluoroborate (2-aminocyclohexanolato-κ2N,O)chlorido(η6-p-cymene)ruthenium(II) Geometric parameters (Å, º)

**Table:**

C1—H1A	0.9800	C21—H21B	0.9900
C1—H1B	0.9800	C21—C22	1.527 (4)
C1—H1C	0.9800	C22—H22A	0.9900
C1—C2	1.498 (4)	C22—H22B	0.9900
C2—C3	1.403 (4)	C22—C23	1.531 (4)
C2—C7	1.443 (4)	C23—H23A	0.9900
C2—Ru1	2.187 (3)	C23—H23B	0.9900
C3—H3	0.9500	C23—C24	1.512 (4)
C3—C4	1.424 (4)	C24—H24	1.0000
C3—Ru1	2.161 (3)	C24—N2	1.486 (3)
C4—H4	0.9500	C25—H25A	0.9800
C4—C5	1.407 (4)	C25—H25B	0.9800
C4—Ru1	2.156 (3)	C25—H25C	0.9800
C5—C6	1.428 (4)	C25—C26	1.505 (4)
C5—C8	1.521 (4)	C26—C27	1.415 (4)
C5—Ru1	2.173 (2)	C26—C31	1.420 (4)
C6—H6	0.9500	C26—Ru2	2.186 (3)
C6—C7	1.397 (4)	C27—H27	0.9500
C6—Ru1	2.178 (3)	C27—C28	1.430 (4)
C7—H7	0.9500	C27—Ru2	2.161 (3)
C7—Ru1	2.184 (3)	C28—H28	0.9500
C8—H8	1.0000	C28—C29	1.411 (4)
C8—C10	1.542 (4)	C28—Ru2	2.183 (2)
C8—C11	1.528 (4)	C29—C30	1.426 (4)
C10—H10A	0.9800	C29—C33	1.518 (4)
C10—H10B	0.9800	C29—Ru2	2.196 (3)
C10—H10C	0.9800	C30—H30	0.9500
C11—H11A	0.9800	C30—C31	1.404 (4)
C11—H11B	0.9800	C30—Ru2	2.181 (3)
C11—H11C	0.9800	C31—H31	0.9500
C12—H12	1.0000	C31—Ru2	2.159 (3)
C12—C13	1.511 (3)	C32—H32A	0.9800
C12—C18	1.518 (4)	C32—H32B	0.9800
C12—O1	1.431 (3)	C32—H32C	0.9800
C13—H13	1.0000	C32—C33	1.533 (4)
C13—C15	1.520 (4)	C33—H33	1.0000
C13—N1	1.479 (3)	C33—C34	1.529 (4)
C15—H15A	0.9900	C34—H34A	0.9800
C15—H15B	0.9900	C34—H34B	0.9800
C15—C16	1.530 (4)	C34—H34C	0.9800
C16—H16A	0.9900	N1—H1D	0.9100
C16—H16B	0.9900	N1—H1E	0.9100
C16—C17	1.530 (4)	N1—Ru1	2.126 (2)
C17—H17A	0.9900	N2—H2A	0.9100
C17—H17B	0.9900	N2—H2B	0.9100
C17—C18	1.532 (4)	N2—Ru2	2.125 (2)
C18—H18A	0.9900	O1—Ru1	2.1129 (18)
C18—H18B	0.9900	O1—H1	0.8401 (10)
C19—H19	1.0000	O2—Ru2	2.1009 (17)
C19—C20	1.524 (3)	B1—F1	1.379 (4)
C19—C24	1.515 (4)	B1—F2	1.394 (4)
C19—O2	1.423 (3)	B1—F3	1.378 (4)
C20—H20A	0.9900	B1—F4	1.382 (4)
C20—H20B	0.9900	Cl1—Ru1	2.4071 (7)
C20—C21	1.526 (4)	Cl2—Ru2	2.4173 (7)
C21—H21A	0.9900		
			
H1A—C1—H1B	109.5	C31—C26—Ru2	69.89 (14)
H1A—C1—H1C	109.5	C26—C27—H27	119.4
H1B—C1—H1C	109.5	C26—C27—C28	121.2 (3)
C2—C1—H1A	109.5	C26—C27—Ru2	71.96 (15)
C2—C1—H1B	109.5	C28—C27—H27	119.4
C2—C1—H1C	109.5	C28—C27—Ru2	71.61 (15)
C1—C2—Ru1	127.27 (19)	Ru2—C27—H27	129.5
C3—C2—C1	121.7 (2)	C27—C28—H28	119.8
C3—C2—C7	117.5 (2)	C27—C28—Ru2	69.96 (14)
C3—C2—Ru1	70.14 (15)	C29—C28—C27	120.3 (2)
C7—C2—C1	120.7 (3)	C29—C28—H28	119.8
C7—C2—Ru1	70.58 (15)	C29—C28—Ru2	71.72 (14)
C2—C3—H3	119.3	Ru2—C28—H28	131.3
C2—C3—C4	121.4 (2)	C28—C29—C30	118.5 (2)
C2—C3—Ru1	72.21 (15)	C28—C29—C33	123.7 (2)
C4—C3—H3	119.3	C28—C29—Ru2	70.69 (14)
C4—C3—Ru1	70.57 (14)	C30—C29—C33	117.9 (2)
Ru1—C3—H3	130.7	C30—C29—Ru2	70.41 (14)
C3—C4—H4	119.5	C33—C29—Ru2	130.49 (18)
C3—C4—Ru1	70.91 (14)	C29—C30—H30	119.6
C5—C4—C3	121.0 (2)	C29—C30—Ru2	71.56 (15)
C5—C4—H4	119.5	C31—C30—C29	120.7 (2)
C5—C4—Ru1	71.69 (14)	C31—C30—H30	119.6
Ru1—C4—H4	130.6	C31—C30—Ru2	70.27 (15)
C4—C5—C6	117.8 (2)	Ru2—C30—H30	131.4
C4—C5—C8	122.8 (2)	C26—C31—H31	119.3
C4—C5—Ru1	70.37 (14)	C26—C31—Ru2	71.95 (15)
C6—C5—C8	119.3 (2)	C30—C31—C26	121.5 (2)
C6—C5—Ru1	71.02 (14)	C30—C31—H31	119.3
C8—C5—Ru1	127.32 (18)	C30—C31—Ru2	71.97 (15)
C5—C6—H6	119.3	Ru2—C31—H31	129.3
C5—C6—Ru1	70.66 (14)	H32A—C32—H32B	109.5
C7—C6—C5	121.3 (2)	H32A—C32—H32C	109.5
C7—C6—H6	119.3	H32B—C32—H32C	109.5
C7—C6—Ru1	71.54 (15)	C33—C32—H32A	109.5
Ru1—C6—H6	131.4	C33—C32—H32B	109.5
C2—C7—H7	119.5	C33—C32—H32C	109.5
C2—C7—Ru1	70.87 (15)	C29—C33—C32	108.2 (2)
C6—C7—C2	120.9 (2)	C29—C33—H33	107.5
C6—C7—H7	119.5	C29—C33—C34	114.4 (2)
C6—C7—Ru1	71.11 (15)	C32—C33—H33	107.5
Ru1—C7—H7	131.3	C34—C33—C32	111.6 (2)
C5—C8—H8	107.6	C34—C33—H33	107.5
C5—C8—C10	109.0 (2)	C33—C34—H34A	109.5
C5—C8—C11	113.7 (2)	C33—C34—H34B	109.5
C10—C8—H8	107.6	C33—C34—H34C	109.5
C11—C8—H8	107.6	H34A—C34—H34B	109.5
C11—C8—C10	111.1 (2)	H34A—C34—H34C	109.5
C8—C10—H10A	109.5	H34B—C34—H34C	109.5
C8—C10—H10B	109.5	C13—N1—H1D	109.9
C8—C10—H10C	109.5	C13—N1—H1E	109.9
H10A—C10—H10B	109.5	C13—N1—Ru1	108.78 (15)
H10A—C10—H10C	109.5	H1D—N1—H1E	108.3
H10B—C10—H10C	109.5	Ru1—N1—H1D	109.9
C8—C11—H11A	109.5	Ru1—N1—H1E	109.9
C8—C11—H11B	109.5	C24—N2—H2A	109.6
C8—C11—H11C	109.5	C24—N2—H2B	109.6
H11A—C11—H11B	109.5	C24—N2—Ru2	110.40 (16)
H11A—C11—H11C	109.5	H2A—N2—H2B	108.1
H11B—C11—H11C	109.5	Ru2—N2—H2A	109.6
C13—C12—H12	108.8	Ru2—N2—H2B	109.6
C13—C12—C18	111.0 (2)	C12—O1—Ru1	112.97 (14)
C18—C12—H12	108.8	C12—O1—H1	112 (2)
O1—C12—H12	108.8	Ru1—O1—H1	127 (2)
O1—C12—C13	106.5 (2)	C19—O2—Ru2	112.03 (14)
O1—C12—C18	112.8 (2)	F1—B1—F2	108.1 (3)
C12—C13—H13	107.8	F1—B1—F4	111.7 (3)
C12—C13—C15	113.3 (2)	F3—B1—F1	111.0 (3)
C15—C13—H13	107.8	F3—B1—F2	107.9 (3)
N1—C13—C12	106.0 (2)	F3—B1—F4	108.7 (3)
N1—C13—H13	107.8	F4—B1—F2	109.3 (3)
N1—C13—C15	113.8 (2)	C2—Ru1—Cl1	157.71 (8)
C13—C15—H15A	109.6	C3—Ru1—C2	37.64 (10)
C13—C15—H15B	109.6	C3—Ru1—C5	69.30 (10)
C13—C15—C16	110.2 (2)	C3—Ru1—C6	80.67 (10)
H15A—C15—H15B	108.1	C3—Ru1—C7	68.11 (10)
C16—C15—H15A	109.6	C3—Ru1—Cl1	155.32 (8)
C16—C15—H15B	109.6	C4—Ru1—C2	69.17 (10)
C15—C16—H16A	109.5	C4—Ru1—C3	38.52 (10)
C15—C16—H16B	109.5	C4—Ru1—C5	37.94 (9)
H16A—C16—H16B	108.1	C4—Ru1—C6	68.15 (10)
C17—C16—C15	110.5 (2)	C4—Ru1—C7	80.78 (10)
C17—C16—H16A	109.5	C4—Ru1—Cl1	117.05 (7)
C17—C16—H16B	109.5	C5—Ru1—C2	82.67 (10)
C16—C17—H17A	109.6	C5—Ru1—C6	38.32 (10)
C16—C17—H17B	109.6	C5—Ru1—C7	68.83 (10)
C16—C17—C18	110.5 (2)	C5—Ru1—Cl1	90.85 (7)
H17A—C17—H17B	108.1	C6—Ru1—C2	68.93 (10)
C18—C17—H17A	109.6	C6—Ru1—C7	37.35 (10)
C18—C17—H17B	109.6	C6—Ru1—Cl1	92.88 (7)
C12—C18—C17	110.1 (2)	C7—Ru1—C2	38.55 (10)
C12—C18—H18A	109.6	C7—Ru1—Cl1	119.36 (7)
C12—C18—H18B	109.6	N1—Ru1—C2	118.08 (9)
C17—C18—H18A	109.6	N1—Ru1—C3	94.14 (9)
C17—C18—H18B	109.6	N1—Ru1—C4	95.31 (9)
H18A—C18—H18B	108.1	N1—Ru1—C5	121.13 (9)
C20—C19—H19	108.8	N1—Ru1—C6	159.28 (9)
C24—C19—H19	108.8	N1—Ru1—C7	156.14 (9)
C24—C19—C20	109.3 (2)	N1—Ru1—Cl1	83.50 (6)
O2—C19—H19	108.8	O1—Ru1—C2	91.15 (9)
O2—C19—C20	113.3 (2)	O1—Ru1—C3	115.85 (9)
O2—C19—C24	107.8 (2)	O1—Ru1—C4	153.72 (9)
C19—C20—H20A	109.6	O1—Ru1—C5	160.23 (9)
C19—C20—H20B	109.6	O1—Ru1—C6	122.04 (8)
C19—C20—C21	110.1 (2)	O1—Ru1—C7	94.75 (9)
H20A—C20—H20B	108.2	O1—Ru1—N1	78.32 (7)
C21—C20—H20A	109.6	O1—Ru1—Cl1	87.82 (5)
C21—C20—H20B	109.6	C26—Ru2—C29	82.01 (10)
C20—C21—H21A	109.4	C26—Ru2—Cl2	163.88 (7)
C20—C21—H21B	109.4	C27—Ru2—C26	37.99 (10)
C20—C21—C22	111.0 (2)	C27—Ru2—C28	38.43 (10)
H21A—C21—H21B	108.0	C27—Ru2—C29	68.88 (10)
C22—C21—H21A	109.4	C27—Ru2—C30	80.77 (10)
C22—C21—H21B	109.4	C27—Ru2—Cl2	148.65 (7)
C21—C22—H22A	109.4	C28—Ru2—C26	69.11 (10)
C21—C22—H22B	109.4	C28—Ru2—C29	37.58 (10)
C21—C22—C23	111.0 (2)	C28—Ru2—Cl2	111.53 (7)
H22A—C22—H22B	108.0	C29—Ru2—Cl2	89.50 (7)
C23—C22—H22A	109.4	C30—Ru2—C26	68.71 (10)
C23—C22—H22B	109.4	C30—Ru2—C28	67.92 (10)
C22—C23—H23A	109.5	C30—Ru2—C29	38.03 (10)
C22—C23—H23B	109.5	C30—Ru2—Cl2	96.25 (7)
H23A—C23—H23B	108.1	C31—Ru2—C26	38.16 (10)
C24—C23—C22	110.7 (2)	C31—Ru2—C27	68.37 (10)
C24—C23—H23A	109.5	C31—Ru2—C28	80.91 (10)
C24—C23—H23B	109.5	C31—Ru2—C29	68.80 (10)
C19—C24—H24	107.8	C31—Ru2—C30	37.76 (10)
C23—C24—C19	112.8 (2)	C31—Ru2—Cl2	125.81 (8)
C23—C24—H24	107.8	N2—Ru2—C26	113.37 (9)
N2—C24—C19	106.9 (2)	N2—Ru2—C27	93.10 (10)
N2—C24—C23	113.4 (2)	N2—Ru2—C28	99.76 (9)
N2—C24—H24	107.8	N2—Ru2—C29	128.37 (9)
H25A—C25—H25B	109.5	N2—Ru2—C30	166.38 (9)
H25A—C25—H25C	109.5	N2—Ru2—C31	149.53 (10)
H25B—C25—H25C	109.5	N2—Ru2—Cl2	82.63 (7)
C26—C25—H25A	109.5	O2—Ru2—C26	93.38 (8)
C26—C25—H25B	109.5	O2—Ru2—C27	122.20 (9)
C26—C25—H25C	109.5	O2—Ru2—C28	160.62 (9)
C25—C26—Ru2	127.87 (18)	O2—Ru2—C29	151.73 (8)
C27—C26—C25	121.0 (2)	O2—Ru2—C30	114.48 (8)
C27—C26—C31	117.8 (2)	O2—Ru2—C31	90.39 (8)
C27—C26—Ru2	70.05 (15)	O2—Ru2—N2	79.09 (8)
C31—C26—C25	121.1 (2)	O2—Ru2—Cl2	87.62 (5)
			
C1—C2—C3—C4	−175.0 (2)	C25—C26—C27—Ru2	−122.9 (2)
C1—C2—C3—Ru1	−122.3 (2)	C25—C26—C31—C30	177.3 (2)
C1—C2—C7—C6	175.1 (2)	C25—C26—C31—Ru2	122.9 (2)
C1—C2—C7—Ru1	122.5 (2)	C26—C27—C28—C29	1.3 (4)
C2—C3—C4—C5	0.0 (4)	C26—C27—C28—Ru2	54.4 (2)
C2—C3—C4—Ru1	53.5 (2)	C27—C26—C31—C30	1.6 (4)
C3—C2—C7—C6	−1.3 (4)	C27—C26—C31—Ru2	−52.9 (2)
C3—C2—C7—Ru1	−53.9 (2)	C27—C28—C29—C30	−1.2 (4)
C3—C4—C5—C6	−1.5 (4)	C27—C28—C29—C33	178.7 (2)
C3—C4—C5—C8	175.5 (2)	C27—C28—C29—Ru2	52.3 (2)
C3—C4—C5—Ru1	53.1 (2)	C28—C29—C30—C31	1.4 (4)
C4—C5—C6—C7	1.6 (4)	C28—C29—C30—Ru2	53.7 (2)
C4—C5—C6—Ru1	54.3 (2)	C28—C29—C33—C32	100.2 (3)
C4—C5—C8—C10	101.0 (3)	C28—C29—C33—C34	−24.8 (4)
C4—C5—C8—C11	−23.5 (4)	C29—C30—C31—C26	−1.6 (4)
C5—C6—C7—C2	−0.2 (4)	C29—C30—C31—Ru2	52.8 (2)
C5—C6—C7—Ru1	52.3 (2)	C30—C29—C33—C32	−79.8 (3)
C6—C5—C8—C10	−82.0 (3)	C30—C29—C33—C34	155.2 (2)
C6—C5—C8—C11	153.5 (2)	C31—C26—C27—C28	−1.4 (4)
C7—C2—C3—C4	1.4 (4)	C31—C26—C27—Ru2	52.8 (2)
C7—C2—C3—Ru1	54.1 (2)	C33—C29—C30—C31	−178.5 (2)
C8—C5—C6—C7	−175.5 (2)	C33—C29—C30—Ru2	−126.3 (2)
C8—C5—C6—Ru1	−122.8 (2)	N1—C13—C15—C16	175.3 (2)
C12—C13—C15—C16	54.1 (3)	O1—C12—C13—C15	−177.9 (2)
C12—C13—N1—Ru1	−47.6 (2)	O1—C12—C13—N1	56.6 (3)
C13—C12—C18—C17	55.9 (3)	O1—C12—C18—C17	175.3 (2)
C13—C12—O1—Ru1	−39.3 (2)	O2—C19—C20—C21	178.6 (2)
C13—C15—C16—C17	−55.3 (3)	O2—C19—C24—C23	178.8 (2)
C15—C13—N1—Ru1	−172.80 (17)	O2—C19—C24—N2	53.5 (3)
C15—C16—C17—C18	58.4 (3)	Ru1—C2—C3—C4	−52.7 (2)
C16—C17—C18—C12	−58.4 (3)	Ru1—C2—C7—C6	52.6 (2)
C18—C12—C13—C15	−54.8 (3)	Ru1—C3—C4—C5	−53.4 (2)
C18—C12—C13—N1	179.7 (2)	Ru1—C4—C5—C6	−54.6 (2)
C18—C12—O1—Ru1	−161.27 (17)	Ru1—C4—C5—C8	122.4 (2)
C19—C20—C21—C22	−58.5 (3)	Ru1—C5—C6—C7	−52.7 (2)
C19—C24—N2—Ru2	−38.8 (2)	Ru1—C5—C8—C10	−169.70 (19)
C20—C19—C24—C23	−57.6 (3)	Ru1—C5—C8—C11	65.8 (3)
C20—C19—C24—N2	177.1 (2)	Ru1—C6—C7—C2	−52.5 (2)
C20—C19—O2—Ru2	−164.26 (16)	Ru2—C26—C27—C28	−54.2 (2)
C20—C21—C22—C23	55.9 (3)	Ru2—C26—C31—C30	54.5 (2)
C21—C22—C23—C24	−53.5 (3)	Ru2—C27—C28—C29	−53.1 (2)
C22—C23—C24—C19	55.3 (3)	Ru2—C28—C29—C30	−53.5 (2)
C22—C23—C24—N2	176.9 (2)	Ru2—C28—C29—C33	126.4 (2)
C23—C24—N2—Ru2	−163.77 (18)	Ru2—C29—C30—C31	−52.2 (2)
C24—C19—C20—C21	58.3 (3)	Ru2—C29—C33—C32	−167.1 (2)
C24—C19—O2—Ru2	−43.1 (2)	Ru2—C29—C33—C34	67.9 (3)
C25—C26—C27—C28	−177.1 (2)	Ru2—C30—C31—C26	−54.5 (2)

### (2-Aminocyclohexanol-κ2N,O)chlorido(η6-p-cymene)ruthenium(II) tetrafluoroborate (2-aminocyclohexanolato-κ2N,O)chlorido(η6-p-cymene)ruthenium(II) Hydrogen-bond geometry (Å, º)

**Table:**

*D*—H···*A*	*D*—H	H···*A*	*D*···*A*	*D*—H···*A*
N1—H1*D*···F3	0.91	2.33	3.150 (3)	150
N1—H1*D*···F4	0.91	2.21	3.032 (3)	150
N2—H2*A*···F2^i^	0.91	2.15	2.996 (3)	154
O1—H1···O2	0.84 (1)	1.61 (1)	2.449 (2)	176 (4)

Symmetry code: (i) *x*, *y*, *z*+1.
